# Dynamic Health Policies for Controlling the Spread of Emerging Infections: Influenza as an Example

**DOI:** 10.1371/journal.pone.0024043

**Published:** 2011-09-06

**Authors:** Reza Yaesoubi, Ted Cohen

**Affiliations:** 1 Center for Communicable Disease Dynamics, Harvard School of Public Health, Boston, Massachusetts, United States of America; 2 Division of Global Health Equity, Brigham and Women's Hospital, Boston, Massachusetts, United States of America; 3 Department of Epidemiology, Harvard School of Public Health, Boston, Massachusetts, United States of America; University of Swansea, United Kingdom

## Abstract

The recent appearance and spread of novel infectious pathogens provide motivation for using models as tools to guide public health decision-making. Here we describe a modeling approach for developing dynamic health policies that allow for adaptive decision-making as new data become available during an epidemic. In contrast to static health policies which have generally been selected by comparing the performance of a limited number of pre-determined sequences of interventions within simulation or mathematical models, dynamic health policies produce “real-time” recommendations for the choice of the best current intervention based on the observable state of the epidemic. Using cumulative real-time data for disease spread coupled with current information about resource availability, these policies provide recommendations for interventions that optimally utilize available resources to preserve the overall health of the population. We illustrate the design and implementation of a dynamic health policy for the control of a novel strain of influenza, where we assume that two types of intervention may be available during the epidemic: (1) vaccines and antiviral drugs, and (2) transmission reducing measures, such as social distancing or mask use, that may be turned “on” or “off” repeatedly during the course of epidemic. In this example, the optimal dynamic health policy maximizes the overall population's health during the epidemic by specifying at any point of time, based on observable conditions, (1) the number of individuals to vaccinate if vaccines are available, and (2) whether the transmission-reducing intervention should be either employed or removed.

## Introduction

The recent appearance of novel human pathogens such as H1N1 and H5N1 influenza, and SARS has stimulated efforts to develop methods to determine health policies that allow for the rapid modification of public health interventions in response to varying epidemiological situations and changing availability of information [1], [2]. In this paper, we examine the potential utility of *dynamic health policies* for controlling the spread of an emerging infectious disease. Dynamic health policies make *real-time* recommendations, in response to changing disease characteristics (e.g. infectivity, antimicrobial resistance levels), population characteristics (e.g. disease prevalence, proportion of individuals that are immune), and resource constraints (e.g. vaccines, antimicrobial drugs, personnel, and budget).

Most existing approaches for identifying optimal strategies for infectious disease control use simulation or mathematical models of disease spread to compare the performance of a *limited* number of *pre-determined* health policies. A number of these studies aim to identify optimal health policies for vaccine allocation *before* the start of an epidemic without explicitly considering interventions which can be employed during the epidemic [3]–[6]. A larger number of studies investigate the effect of both initial immunization and the use of controlling interventions during epidemics, such as use of antiviral for treatment, case isolation, school closure, and internal travel and border restrictions [7]–[13].

Although these approaches can provide insight into which baseline strategies may best reduce the impact of epidemics, they are not generally structured to assist real-time decision making through the *dynamic* change of health recommendations as new data become available over the course of epidemic. In this paper, we focus on developing optimal dynamic health policies for controlling an emerging human pathogen. These policies allow decision-makers to use cumulative real-time data from the epidemic and current information about resource availability to guide their selection of possible interventions at any particular point in time.

We use a simplified model of influenza spread to illustrate the development and the employment of dynamic health policies. Control of influenza epidemics may involve: (1) reducing the susceptibility of uninfected individuals either before or during the epidemic (through vaccination or antiviral prophylaxis), (2) reducing contact rates in the population (through social distancing such as isolation of diagnosed cases, quarantine of households of diagnosed cases, closing of schools), and (3) reducing the infectiousness of infected individuals (through treatment or isolation).

While vaccines provide protection from seasonal influenza, the emergence of a novel strain would likely mean the absence (or shortage) of an effective vaccine for at least the first several months of the epidemic [14], [15]. During this period, control strategies would largely rely on social distancing and potentially on stockpiles of antiviral drugs used for treatment and/or prophylaxis. Therefore, efforts to control an emerging influenza epidemic would be bounded by (1) the availability of effective vaccines and antiviral drugs, and/or (2) the availability of money and resources for vaccine procurement, diagnosis and treatment of new cases, and the actual implementation of transmission reducing interventions during the epidemic. Under such resource constraints, we define the *optimality* of a dynamic health policy as the efficient use of available resources (e.g. budget and medical system capacity) to maximize the overall health of the population (e.g. minimizing the number of deaths or hospitalizations, or maximizing other measures such as quality-adjusted life years).

In this paper, we define several broad types of interventions for controlling the spread of an emerging influenza virus. We use these crudely classified interventions and a simplified model of influenza transmission to illustrate the use of a dynamic optimization methodology (namely, Markov Decision Process [16]) to specify dynamic health policies. Here, rather than focusing on developing a comprehensive model of influenza spread, we demonstrate how these dynamic policies allow real-time decision making under different resource constraints. We also discuss how future research in this area can help make the implementation of these policies possible.

## Method

In this section, we first discuss the set of possible interventions that can be used for controlling an influenza epidemic. We describe the effect of these interventions on disease spread as well as on the overall health-related and monetary consequences of the epidemic. We then define dynamic health policies for controlling the epidemic and characterize their optimality. We finally propose a methodology to identify the optimal dynamic health policies for controlling the epidemic.

### A Model for Controlling the Spread of Influenza

#### Influenza Epidemic State and Decision Sets

An influenza epidemic is usually described by a SIR (Susceptible-Infective-Recovered) model, in which the individuals recovered from infection are assumed to acquire permanent immunity to that viral strain [7], [17]. Let 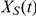 denote the number of susceptibles, 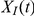 denote the number of infectives, and 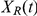 denote the number of recovered at time 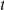. Since influenza epidemics usually last for several months and the number of deaths is generally small relative to population size, it is reasonable to assume that the population size does not change over the course of epidemic. For a population of a fixed size 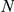, the state of the disease spread at any given time 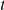 can be identified by 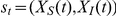. Let 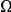 denote the state space defined as 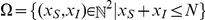.

Decisions are made at points of time referred to as *decision epochs*. It is more convenient to assume that the decisions are made at discrete points of time, rather than continuously over time; hence, we assume that the set of decision epochs, 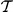, is discrete; that is 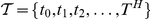, where 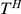 denotes the decision horizon length. We classify the possible interventions to control the spread of influenza into two categories: (1) “irreversible” interventions such as vaccination employed either before or during the epidemic which reduce the number of susceptibles, and (2) “reversible” interventions which can be turned on and off during the course of epidemic to reduce the transmission of infection to susceptibles, such as hygienic interventions, social distancing, and treatment. We implement these two types of decisions in our model as follows:

1. Vaccination: At any decision epoch, conditional on the availability of vaccine, the decision maker will specify the number of susceptibles to vaccinate. If effective vaccines are abundant and vaccination has zero cost, this decision is trivial: vaccinate all susceptibles. However, throughout this paper we assume that vaccine, if available, is acquired at a price. We denote this decision by 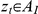, where 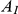 is the set of possible values for the decision variable 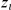. For cases where vaccine availability exceeds need, we may assume that 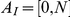. For simplicity, we assume that vaccination at decision epoch 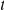 results in immunization by the next decision epoch. Hence, a decision to vaccinate 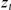 portion of susceptibles at time 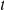 results in the reduction of susceptibles to 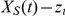 by the next decision epoch.
2. Transmission-reducing intervention: These interventions may be either employed or lifted over the course of epidemic to reduce the transmission of infection to remaining susceptibles. These measures will include social distancing (e.g. school or public place closure), hygienic interventions (e.g. mask use), and treatment or isolation of cases. Let 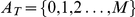 denote the set of such interventions, where 0 represents “no intervention.” We denote the transmission-reducing decision made at time 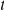 by 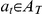.

We categorize “treatment” as a transmission-reducing intervention which can be turned on or off during the epidemic. Of course, when sufficient antivirals are available, all new cases receive appropriate treatment, in which case “treatment” will not be included as a decision in the model. However, under conditions of antiviral limitation, one may include “treatment” as an intervention which can be turned on or off during the epidemic; this situation may occur when the use of antiviral must be prioritized among population subgroups [3], [4].

To control the epidemic, a policy maker will continue to make decisions until the prevalence of the disease is sufficiently low. The stochastic nature of transmission (which is especially important during emergence or eradication), prevents accurate identification of the time when the disease will be eradicated; hence, we consider an infinite decision horizon: 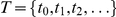 [16]. Although the decision horizon is infinity, we assume the decision making process stops when there are no more infectious individuals in the population.

#### Rewards and Transition Probabilities

As the result of vaccination employed at time 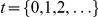, the number of susceptibles is reduced from 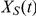 to 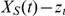 by the next decision epoch, and the policy maker receives a reward 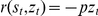, where 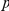 is the unit price of vaccine.

As the result of a transmission-reducing intervention employed at time 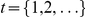, i.e. 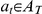, the spread of disease at the next decision epoch is determined by the probability distribution 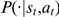, and the policy maker receives a reward 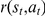.

The reward 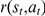 can be characterized in several ways and the choice of reward structure should reflect the policy maker's set of priorities. For example, if the policy maker wants to minimize the total number of individuals infected over the course of epidemic, then reward 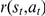 can be simply defined as 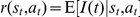, where 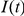 is the number of new infections during the period 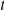. However, efforts to control epidemics may be bounded by the availability of medical resources, such as vaccines, medical personnel, and antiviral drugs, and monetary resources for vaccine procurement, diagnosis and treatment, and implementation of interventions. In these situations, where both health-related outcomes and the resource consumption level are essential for determining the optimality of a health policy, a more comprehensive reward function is needed. A common approach for defining optimality in these situations is to assume that the policy maker's objective is to maximize the population's net monetary benefit [18]. To characterize the reward 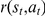 accordingly, we must define several additional parameters:

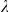
: policy maker's willingness-to-pay (WTP) for health.

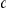
: cost incurred for each incident infection (this may include diagnosis, treatment and other indirect costs).

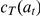
: cost of implementing the intervention 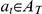 for period 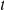; we assume 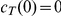.

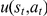
: expected costs incurred during the period 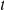 if the disease spread at time 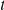 is at state 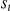 and the policy maker implements the intervention 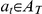 at decision epoch 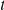; 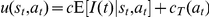.

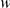
: loss in health (quantified by quality-adjusted life years) due to infections.

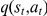
: expected loss in population's health during the period 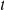 if the disease spread at time 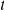 is at state 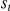 and the policy maker uses intervention 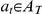 at decision epoch 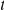; 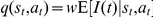.

Now, the reward 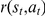, defined as the expected net monetary benefit during the period 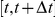 if the disease spread at time 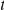 is at state 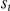 and the policy maker chooses intervention 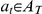 at decision epoch 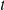, is calculated by 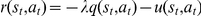.

#### Decision Rules, Health Policies and Optimality

A *decision rule* prescribes an action for each state for a specified decision epoch. For decision epoch 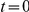, a decision rule is a function 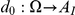, which specifies the number 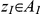 of susceptibles to vaccinate given the initial disease state 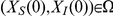. For decisions that must be made during the course of the epidemic (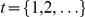), we focus on a *Markovian* decision rule because this is the most convenient to implement and evaluate. Such decision rules are functions 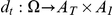, which for each state of the disease spread, assign a transmission-reducing intervention 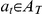 and a proportion 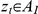 of susceptibles to vaccinate. This decision rule is said to be *Markovian* (memoryless) because it depends on previous disease states and previously utilized interventions only through the current state of the disease spread.

A *policy* specifies a decision rule to be used at all decision epochs. In other words, a policy 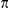 is a sequence of decision rules 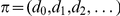. We call a policy *stationary* if 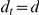 for 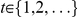; that is, the policy prescribes the same decision for the given state 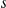 regardless of the time period in which this state is reached. In this paper, we are only interested in characterizing stationary health policies since they are the most feasible to implement in practice and their optimality can be proven [16].

Assuming that the influenza spread is at state 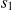 at decision epoch 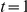, the expected total discounted reward induced by policy 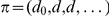 over the course of epidemic is calculated as:
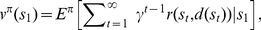
(1)where 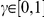 is the discount factor to account for the time value of the future rewards.

Now, assuming that at decision epoch 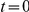, the state of the disease spread is 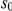, the expected total reward induced by policy 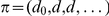 is:
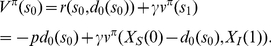
(2)


Let 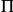 denote the set of all possible stationary policies 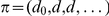. We say that a policy 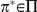 is optimal whenever:
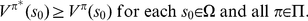
(3)


Define 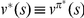, where 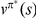 is calculated by Eq. (1) for 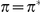.

Implicit in the definition of stationary policies is the assumption that model parameters are known and do not change over time. This assumption allows the existence of an optimal stationary policy 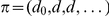, where the function 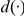 is time independent. Undoubtedly, this assumption may be violated for emergent influenza epidemics, and the model parameters may need to be updated as new data accrues over time. We return to this issue in later sections and discuss how stationary policies can be determined in this setting. But, for clarity of presentation, we first assume that the parameters of the influenza model are constant and estimable from the initial spread of the epidemic.

### A Markov Decision Process Formulation for Influenza Spread

There are several dynamic optimization methodologies that can be used to find the policy 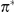 defined in inequality (3). The most appropriate optimization method depends on the complexity of the underlying epidemic model, the observability of the epidemic state, and the desired level of computational efficiency. As a rule of thumb, finding the *exact* optimal policy 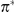 becomes more challenging (and sometimes impossible) as (1) the complexity of epidemic model increases or (2) the uncertainty around the true state of the disease spread arises. Several methodologies can help identify the *approximate* optimal policy 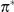 even when the epidemic model is relatively complex and only some probabilistic knowledge about the current state of the epidemic can be obtained. We discuss the challenges of optimal decision-making under uncertainty at further length in the Discussion section.

If the disease dynamics can be modeled by a discrete-time Markov chain and the state of the epidemic is observable over the course of epidemic, then the stationary optimal health policy 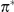 in inequality (3) can be efficiently obtained through Markov Decision Process (MDP) [16]. In our illustrative example of a novel influenza epidemic, we make several simplifying assumptions about the spread of influenza to be able to use MDP. We enumerate these assumptions in the following subsections; we then discuss how these simplifying assumptions can be relaxed in further work.

We describe epidemic influenza with a SIR model, in which individuals acquire permanent immunity through infection or vaccination. We do not consider the possibility of changes in the population size due to birth, immigration, or death in order to simplify the analysis. Let 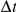 denote the time interval between two consecutive decision epochs. We assume that individuals become infected only through contact with other infectious members of the population, and that contacts during the interval 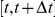 occur according to a homogenous Poisson process, with rate 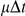.

We assume that a susceptible who is infected during period 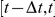 becomes infectious and symptomatic at time 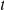 and interacts with the rest of the population during period 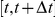; the individual is then removed from the population (or recovered) at time 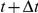. As we will describe later, these simplifying assumptions allow the use of Markov decision process to identify the optimal decision at each decision epoch. In the Discussion section, we explain how these assumptions can be relaxed.

Let 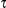 denote probability that a susceptible person becomes infected upon contact with an infectious individual and 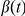 denote probability that the next interaction of a random susceptible person is with an infectious person. When social distancing has not been used and mixing is homogenous, 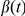 is equal to 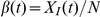. Variables 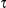 and 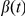 can be respectively modified by “hygienic interventions” (reducing the chance of transmission given contact between infectious and susceptible individuals) and “social distancing” (reducing the likelihood of contact between susceptible and infectious individuals). Let 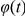 denote overall probability that a susceptible person becomes infected. This probability is calculated in [19] as:
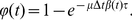
(4)


Hence, given the state of the disease spread, i.e. 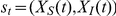, the number of new infections during period 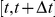, denoted by 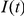, will have a binomial distribution with number of trials 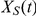 and the probability of success 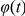:
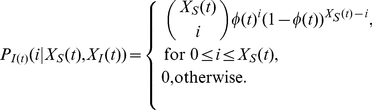
(5)


The transition probabilities of the Markov chain 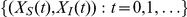 can then be calculated by (refer to Text S1 for detailed steps):
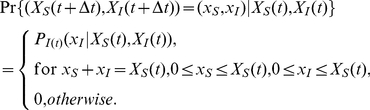
(6)


We consider two types of interventions: (1) vaccination and (2) transmission-reducing interventions. First, we assume that no vaccines will become available any time during the epidemic; therefore, the health policy 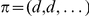 only specifies the optimal transmission-reducing intervention 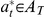 to implement at decision epoch 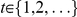. We will show later how optimal vaccination decisions can be made once vaccine becomes available. Also, for simplicity, we assume that only one transmission-reducing intervention is available; hence 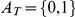. Selecting intervention 1 at decision epoch 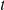, i.e. 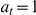, reduces probability 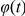 in Eq. (4) to 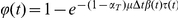, where 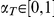 is the fractional reduction in the infection transmission rate.

Let 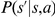 denote the probability that the influenza epidemic will be in state 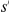 at decision epoch 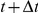, given that that the state is 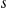 at decision epoch 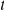 and the policy maker implements intervention 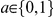 at decision epoch 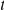. This probability is calculated by transition probability (6).

Since we first assume that no vaccine is available during the course of the epidemic (we relax this assumption below), the policy maker can only select decisions from set 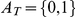. Given this assumption, the optimal solution to the policy maker's problem during the course of epidemic (i.e. maximizing Eq. (1)) is obtained by solving the following set of recursive equations [16]:
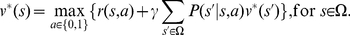
(7)


By definition, function 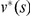 returns the expected total discounted reward induced by the optimal policy over the remaining course of epidemic if the current state of epidemic is 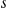. Therefore, having found the function 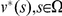, by solving the set of equations (7), we can then determine the optimal transmission-reducing decision for a given state 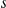 by:
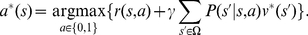
(8)


## Results

To illustrate the use of the proposed methodology, we consider the case of an influenza outbreak in an English boarding school reported in [20] and recently used by [2] and [21]. The population consisted of 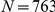 students and the infection was believed to be introduced by one student returning from Asia. The situation satisfies many requirements of a simple SIR model, particularly since no specific intervention was employed during the outbreak.

For a population of size 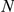, the transition probability matrix of the Markov chain 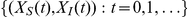 is of size 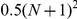, which causes computational problems for our effort to identify optimal health policies. To overcome this computational difficulty, several effective alternative solutions have been proposed in the literature of dynamic optimization; the reader is referred to [22] for comprehensive discussion. One approach to reduce the state space of the Markov chain 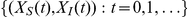 is “state aggregation”, in which the Markov chain 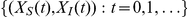 is approximated by the Markov chain 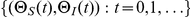, where 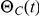, 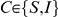, is the *proportion* of population in class 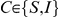 at time 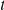. Detailed steps for how one can make these approximations are provided in the attached Text S1. Note that although we consider a relatively small population here, however, the approximation method briefly described above can also be used for larger populations.

**Figure 1:**
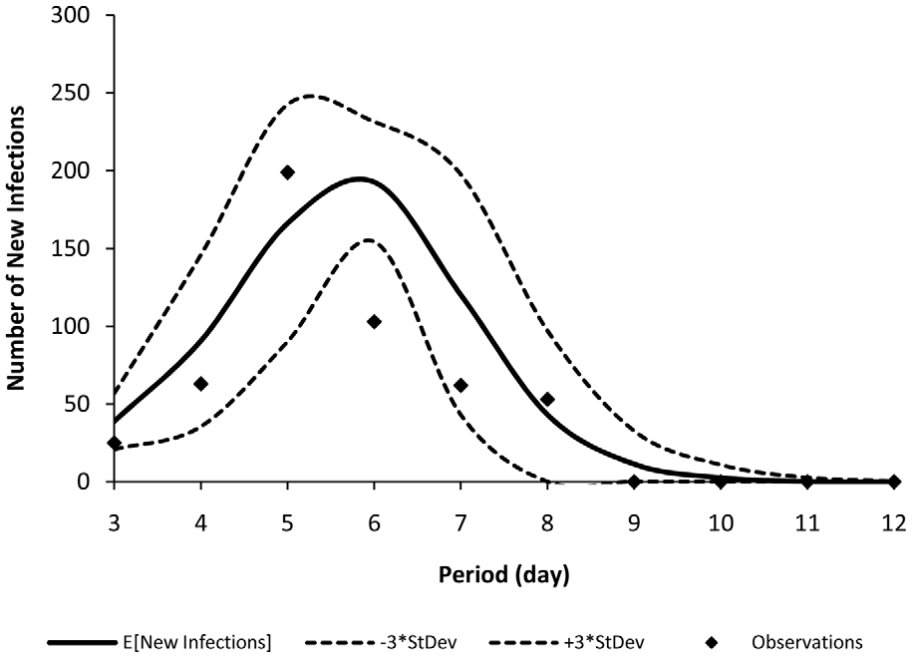
Observed new cases in the English boarding school versus the model's predictions. This figure shows each day's expected number of new infections calculated by the model (presented by solid curve) versus the observed data (presented by dots). The dotted curves show the model's expected number of new infections 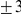 times its standard deviation.

The influenza spread model described previously has two parameters, 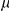 and 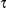, which should be estimated from the data. Using maximum likelihood estimation, we estimate 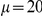 per day and 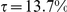. Figure 1 shows each day's expected number of new infections calculated by the model versus the observed data. Note that in Figure 1, the observations on days 1 and 2 are not shown. The reason is as follows: since in the approximate Markov chain 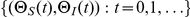, the states of influenza spread are aggregated, the model is not able to accurately capture the state of the epidemic when the number of infections is very low. For the observations presented in Figure 1, the number of new cases in days 1 and 2 are respectively 1 and 6, which are too low to be captured by the approximate Markov chain 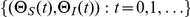. For detailed discussion, refer to Text S1 and [19].

To determine optimal dynamic health policies for this population, we use the following arbitrary settings. We consider one transmission-reducing intervention which reduces the rate of infection transmission by 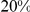, i.e. 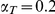, and costs 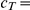 $.,2000 per day. One such transmission-reducing intervention might be “having all students wash their hands twice a day”. The vaccine price is set to 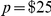; each incident infection costs 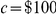 to diagnose and treat and results in a health loss of 0.00342 QALY (assuming that the treatment period lasts 5 days during which the health quality of the patient is reduced by 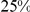: 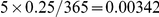).

Optimal policies during the epidemic are obtained by solving the set of equations (7) for 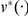 and then using Eq. (8) to find the optimal decision 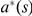 for each state 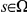. We use a policy iteration algorithm [16] with a discount factor 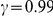 to solve the set of equations (7).

**Figure 2:**
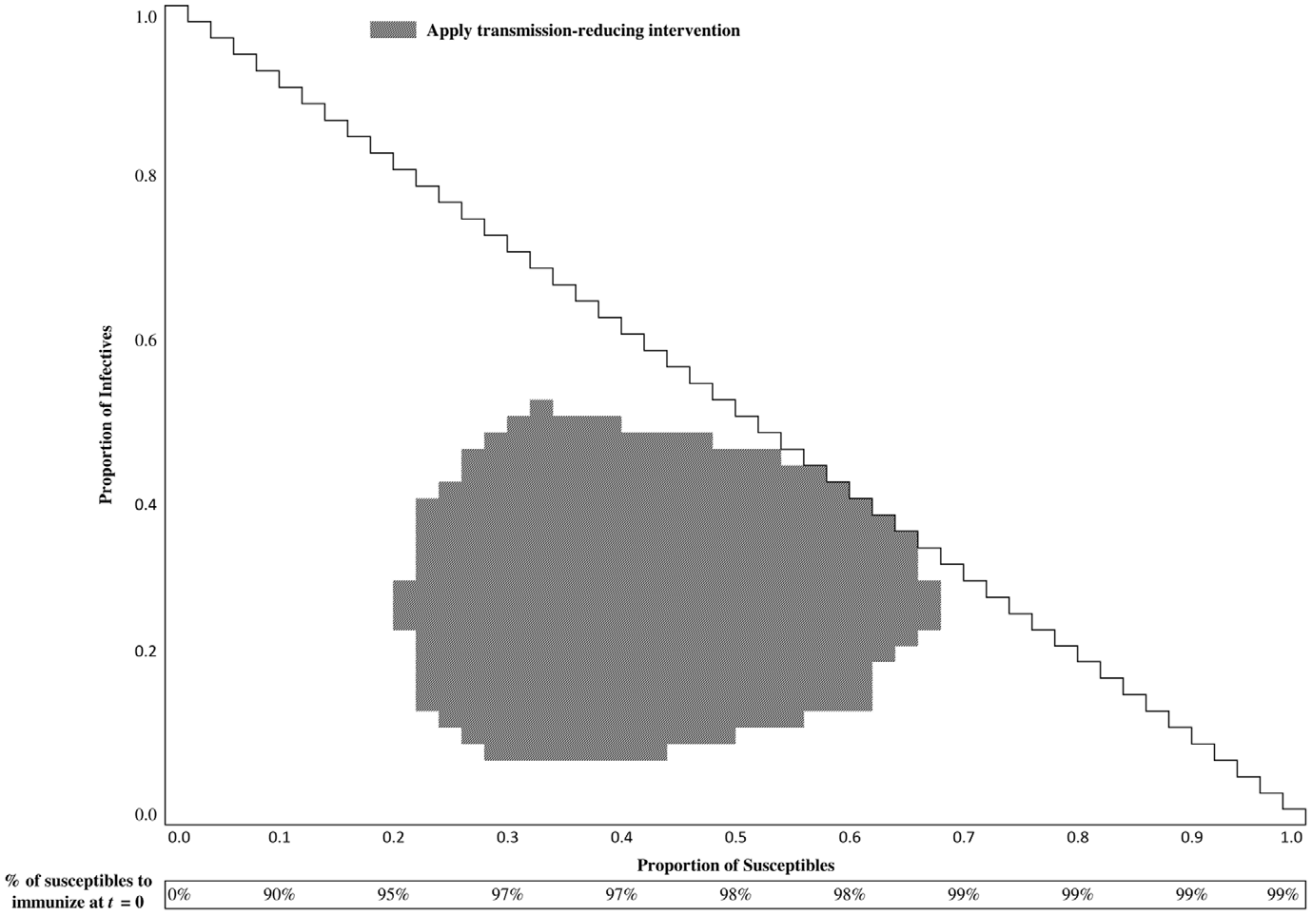
Optimal stationary health policy for 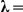
$.,25000/QALY. The conditions for recommending the use of the transmission-reducing intervention is presented within the triangle in the upper part of each figure. In disease states consistent with those that are captured within the grey cells, the intervention should be used. For instance, the transmission-reducing intervention should be implemented if the state of the disease is 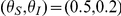, and should not be used when the state of the disease is 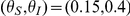. The rectangle in bottom of this figure, labeled as “% of susceptibles to immunize at 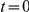,” represents the optimal health policies for immunization before the start of epidemic given that vaccine is available at a market price.

Figure 2 displays the optimal health policies for WTP for health equal to $.,25000/QALY. The conditions for recommending the use of the transmission-reducing intervention are presented as the grey regions within the triangle in the upper part of each figure. For example, the transmission-reducing intervention should be implemented if the state of the disease is 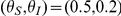, and should not be used when the state of the disease is 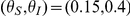. Text S1 includes additional health polices corresponding to different levels of WTP.

As discussed previously, we must also identify the optimal number of susceptibles to vaccinate when an effective vaccine becomes available. Let us assume that the entire population is susceptible before the start of epidemic, i.e. 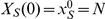, among which 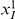 susceptibles become infectious by time 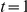. If we assume that effective vaccines become available at time 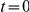 at a market price 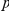, then the policy maker determines the number the suscpetibles to immunize, 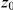, by solving the following optimization problem:
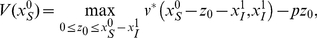
(9)in which, the variable 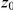 only takes integer values over the interval 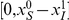.

The rectangle in bottom of Figure 2, labeled as “% of susceptibles to immunize at 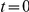,” represents the optimal health policies for immunization before the start of epidemic; again, if vaccine is not yet available, it may not be possible to achieve these levels of immunization. These recommendations are obtained by solving problem (9).

### Employing Dynamic Health Policies to Control Influenza Spread

In this section, we discuss how the optimal dynamic health policy for using the transmission-reducing intervention and vaccinating additional susceptible individuals in decision epochs is determined as an epidemic progresses and new data become available.

Table 1 shows the observable information that accrues during an epidemic. Before the start of epidemic, at time 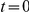, the policy maker obtains an estimate for 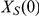, denoted by 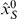. For a novel strain of influenza, we assume that the entire population is susceptible, hence 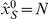 for these situations. The policy maker also obtains a prior distribution for the number of susceptibles who may become infectious by time 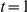. Let the number of infectives at time 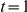 be randomly distributed according to probability mass function 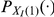 with support 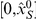. The policy maker can then use the following optimization problem to find the number of susceptibles to immunize at time 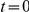:
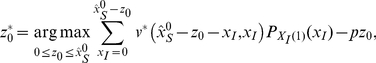
(10)in which, the variable 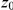 only takes integer values over the interval 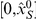.

**Table 1:** Observed information over the course of an epidemic.

Period	0	1	2	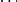	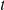
Observed number of	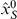	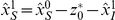	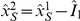		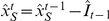
susceptibles, 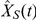					
Observed number of		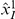	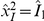		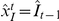
infectives, 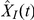					
Observed new infections, 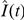		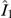	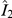		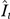

Using observation of the number of new infections occurring during the epidemic, we update our knowledge on the state of the epidemic for the next decision epochs as follows. At the beginning of period 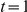, 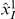 new infections are observed which implies that the number of susceptibles at this time is 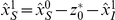. At the end of each period 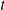, 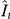 new infections are observed. This observation implies that (1) the number of susceptibles at the beginning of period 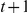 is 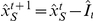 and (2) the number of infectives at time 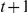 is 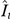, since we assume that all infectives at time 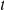 are removed or recovered by the next decision epoch (see Table 1).

Now, knowing that the epidemic is at state 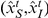 and assuming that no vaccine is available at time 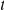, the optimal transmission-reducing decisions for period 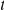 is obtained by solving Eq. (8) (which can also be summarized in form of the policy shown in Figure 2).

Now assume that at decision epoch 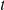, vaccines for the epidemic strain of influenza either become available for the first time or that depleted stocks of vaccine have now been replenished. Let 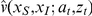 denote the expected reward if influenza spread is in state 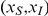 at time 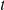, the transmission-reducing intervention 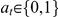 is implemented during period 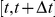, and 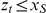 susceptibles are vaccinated by the decision epoch 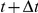. Then, function 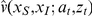 is calculated as:
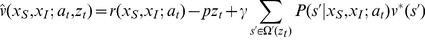
(11)where 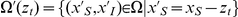. The optimal transmission-reducing intervention (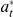) and the number of susceptibles to vaccinate (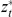) during the period 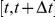 is then determined by solving the following problem:
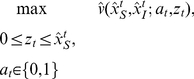
(12)where 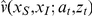 is given by Eq. (11) and the variable 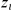 only takes integer values over the interval 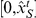.

As briefly pointed out before, to determine the stationary health policy 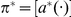, we assumed that the parameters of the influenza spread model are all known and do not change over time. It is, however, more realistic to assume that as new data become available over the course of epidemics, policy makers also update the parameters of the underlying transmission model. Table 2 outlines a procedure describing how dynamic health policies can be employed when policy maker is using the real-time data to also update the parameters of the influenza spread model.

**Table 2:** Determining Dynamic Health Policies Using Real-Time Data.

**At time** *t* = 0
1. Obtain estimates for model parameters ( 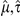 ), initial number of susceptibles, 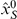 , and a prior distribution for random variable 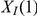 .
2. Use Eqs. (7)–(8) to determine the dynamic health policy 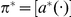 .
*3*. Find the optimal number of susceptibles to immunize, 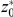 , using Eq. (10).
4. Increment time to 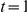 .
*5*. Update the number of susceptibles at time 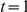 : 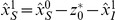 .
**While** 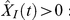
1. Given the availability of vaccine, use Eq. (8) or Eq. (12) to find the optimal number of susceptibles to immunize, 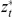 , and the optimal transmission-reducing intervention, 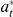 .
2. At the end of period 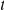 , use the real-time data 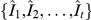 to update the model parameters (using for instance, maximum likelihood estimation) and the dynamic health policy 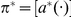 , using Eqs. (7)–(8).
3. Increment time: 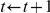 .
4. Use Table 1 to update the epidemic state at time 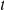 .

### Optimal Dynamic Health Policies under Resource Constraint

The optimal health policies developed in the previous section do not consider the availability of resources (e.g. vaccines, budget, and antiviral for treatment) while making health recommendations. For example, the policy presented in Figure 2, recommends vaccinating 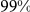 of the remaining susceptibles when the initial proportion of susceptibles is 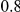; this recommendation does not account for the number of vaccines that are actually available. Similarly, the recommendations for turning on and off the transmission-reducing intervention do not account for the budget and resources necessary to initiate or terminate these recommendations. The framework proposed here can be expanded to incorporate different forms of resource constraints while constructing dynamic health policies. In this section, we discuss policy development under three types of resource limitations.

**Figure 3:**
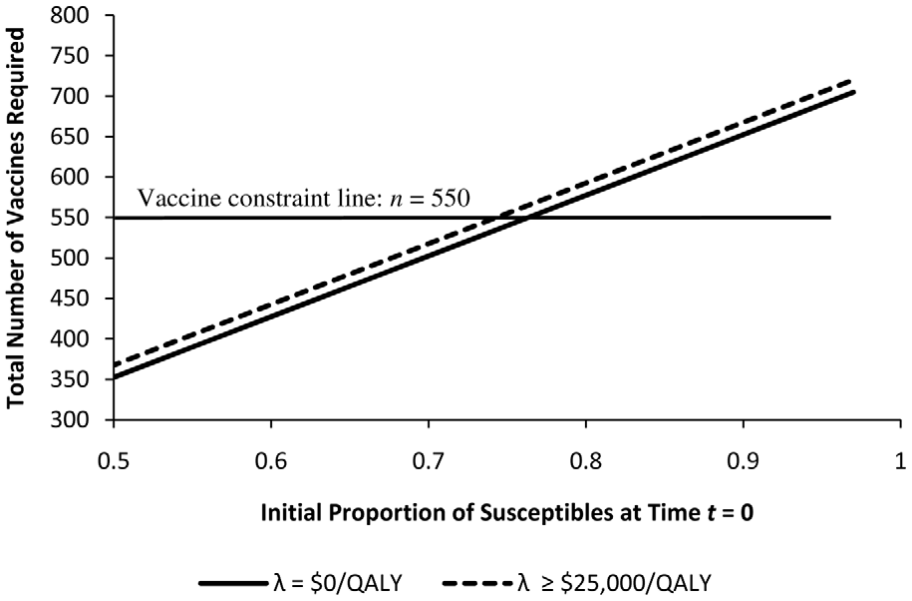
Number of Vaccines Required. Each line specifies the optimal number of vaccines required by different WTP for health. For a given initial number of susceptibles, the policy maker can use this figure to find the WTP for health whose corresponding policy satisfies the vaccine constraint.

Let us first consider the case where 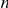 vaccines are available at time 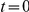, and the policy maker must decide how many susceptibles to vaccinate using these 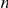 vaccines. For now, let us assume that no additional vaccines will become available during the epidemic and any unused vaccine by time 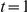 will be lost (e.g. shipped to other communities). The policy maker can now use Figure 3 to select the WTP for which a feasible vaccination recommendation can be implemented. For a given initial proportion of susceptibles 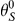, Figure 3 identifies the number of vaccines required for each value of WTP (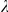) through solving the optimization problem (10). As an example, for 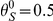, if the policy maker's sole objective is to minimize cost (i.e. 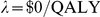) 352 vaccines are required and for any WTP 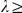 $.,25000/QALY, 367 vaccines should be used. Note that for 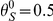, vaccinating less than 352 susceptibles results in a monetary loss, and vaccinating more than 367 susceptibles does not increase the population's expected net monetary benefit for any 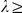 $.,25000/QALY.

Now to demonstrate how a vaccine supply constraint can be accounted for in constructing an optimal health policy, let us assume that for our population, at time 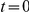 (before the start of epidemic), there are 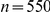 vaccines available (the same approach can be followed for all other decision epochs). When 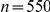 vaccines are available, then according to Figure 3, for any initial proportion of susceptibles 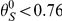 any health policy with 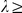 $.,25000/QALY can be used to optimally allocate all of the available vaccines. For any initial proportion of susceptibles 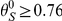, all 550 vaccines should be used.

Now we consider a more complex scenario of vaccine limitation where vaccines become available in varying quantities over several decision epochs. Let us assume that at time 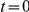, the policy maker knows with certainty that during the following 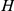 decision epochs 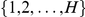, 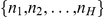 vaccines will become available. Like before, we assume that any vaccine unused during a period will be lost at the end of the period. Given epidemic state 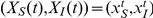 and 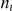 available vaccines at time 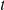, the policy maker must now decide how many susceptibles to vaccinate and also whether to implement the transmission-reducing intervention. To illustrate how these decisions can be made at each decision epoch, let us assume that 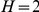 (cases with 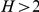 are solved in a similar fashion).

The optimal allocation is determined iteratively as follows. We start by finding the optimal decisions for the final decision epoch for which vaccines are supplied (i.e. 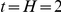 in this example). If at decision epoch 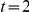, the epidemic is at state 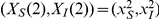 with 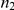 vaccines available, the optimal recommendations for vaccination and transmission-reducing intervention are determined by solving the problem:
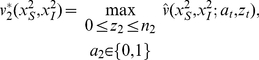
(13)where 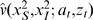 is calculated by Eq. 11. Next, we step backward in time and determine the optimal decisions for epoch 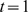. If at time 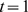, the epidemic is at state 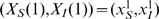 with 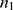 vaccines available, the optimal decisions at time 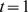 is determined by:
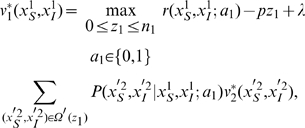
(14)where 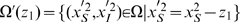 and 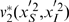 is already calculated in problem (13).

Now to employ this new dynamic health policy that was generated under assumptions of known vaccine constraints 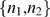, we take the following steps (note that we assume here that no vaccine is available at time 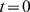):

Step 1: At time 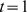, for the observed state 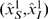 and 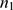 available vaccines, use problem (14) to find the optimal number of susceptibles to vaccinate and the transmission-reducing intervention to implement.
Step 2: Update the epidemic state using the observed number of new cases during period 1, 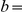 (see Table 1).
Step 3: At time 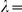, for the observed state 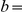 and 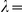 available vaccines, use problem (13) to find the optimal number of susceptibles to vaccinate and the transmission-reducing intervention to implement.
Step 4: For the remaining decision epochs 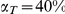, use Table 1 to update the epidemic state and then use problem (8) to find the optimal transmission-reducing intervention to employ.

**Figure 4:**
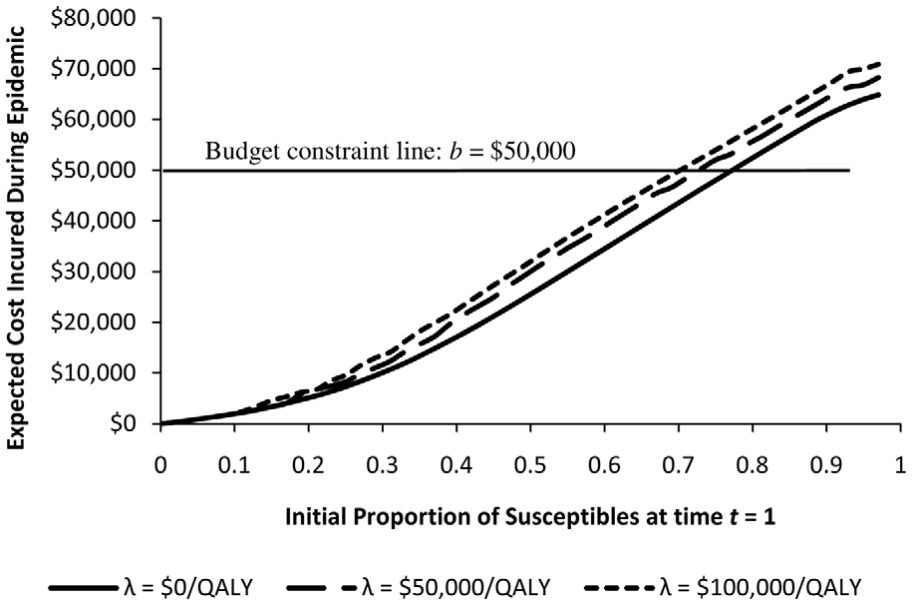
Expected Budget Required During Epidemic. Each curve specifies the expected budget required by different WTP for health. For a given initial number of susceptibles, the policy maker can use this figure to find the WTP for health whose corresponding policy satisfies the budget constraint.

Finally, we discuss how budget constraints can be accounted for while generating dynamic health policies. From a methodological perspective, ensuring that budgetary limitations are not exceeded while attempting to control an epidemic is more challenging primarily due to the stochastic nature of disease spread that leads to high variance for the expected costs incurred. However, if we assume that the policy maker is mainly interested in keeping the *expected* cost incurred during epidemic lower than a constant budget threshold, Figure 4 can be used to select the health policy which satisfies such a constraint.

Suppose that for our population, after vaccination phase at 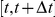, the proportion of susceptibles at 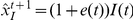 is reduced to 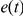, and that the policy maker sets the budget threshold at 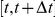
$.,50000. Then, Figure 4 indicates that for WTP 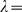
$.,100000/QALY the expected cost incurred during epidemic remains below 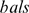
$.,50000. Therefore, the policy maker should use the health policy generated by setting the willingness-to-pay 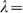
$.,100000/QALY to guide decision making during the epidemic.

### Evaluating the Effect of Dynamic Health Policies in Controlling the Spread of Influenza

**Figure 5:**
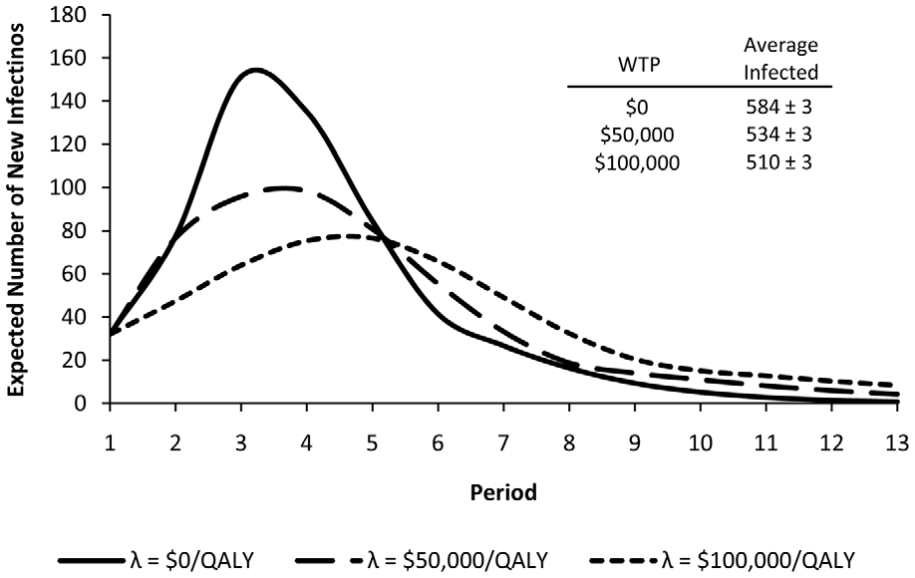
Effect of dynamic health policies on controlling the spread of influenza when no vaccine is available. As the willingness-to-pay for health increases, the expected number of individuals infected during the epidemic is reduced since the policies corresponding to the higher willingness-to-pay tend to be more aggressive in implementing the transmission-reducing intervention.

To study the effect of employing the dynamic health policies, we built a simulation model for influenza spread in the population described above. Figure 5 displays the expected number of new infections during each period when 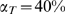 and policies corresponding to different WTP are employed, given the realistic assumption that no effective vaccine was available before the start of epidemic. If vaccines were available in sufficient quantities to employ the and the vaccine recommendations in Figure 5, the epidemic would be averted and there would be no need to employ dynamic policies. The incidence of disease for these policies in each period were statistically different with each other at 1000 simulation runs. As shown in Figure 5, as the willingness-to-pay for health increases, the expected number of individuals infected during the epidemic is reduced since the policies corresponding to the higher willingness-to-pay tend to be more aggressive in implementing the transmission-reducing intervention.

### Evaluating the Assumption of the Observability of New Cases

In previous section, we assumed that the policy maker is able to accurately measure the number of new infections occurring in each period. This is a strong assumption that will be violated for many infectious diseases (including influenza) where diagnosis is difficult and for which the number of reported cases is an underestimate for the actual number of infections. To examine the sensitivity of the performance of the generated dynamic health policies to the assumption that all cases are observed, we assume that the policy maker's observation of the number of cases during period 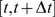 is only a portion of the true number of cases; that is 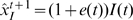, where 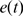 is the percent error in identifying the number of new cases during period 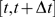.

If the error term 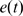 is a constant in each period, i.e. 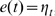, and known to the policy maker, the number of susceptibles and the number of infectives in Table 1 can be calculated, respectively, as 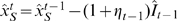 and 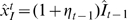. Hence, the constant observation error 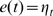 can be easily corrected in order to preserve the optimality of dynamic health policies under this error setting.

**Figure 6:**
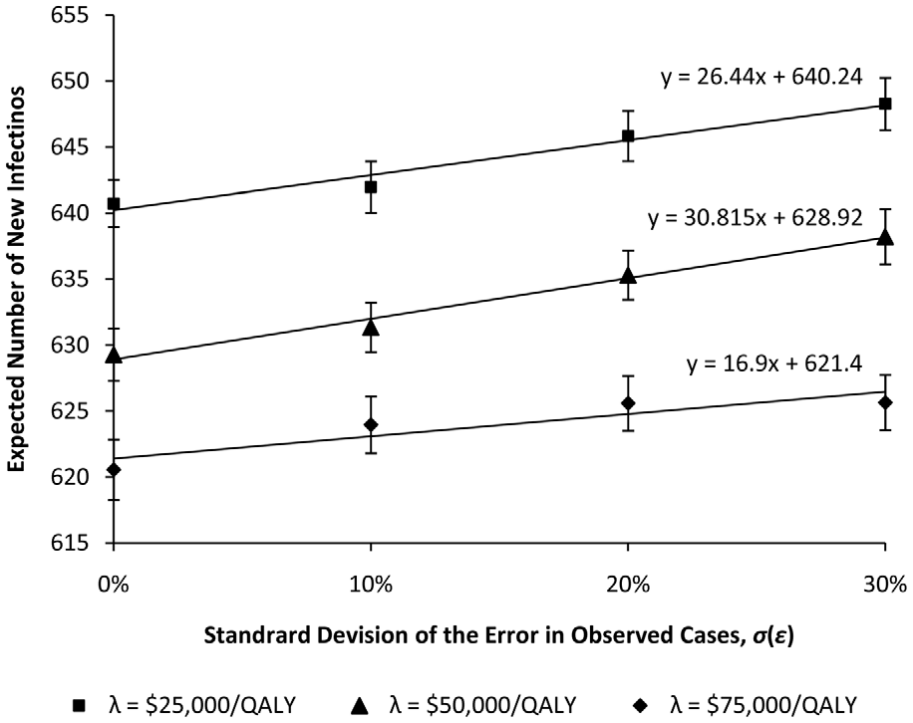
Expected total number of new infections versus the standard deviation of the error in observing the number of new cases. As the standard deviation of noise increases, reflecting imperfect surveillance capacity, the total number of individuals affected by the epidemic also increases. However, this increase occurs linearly at a low rate, which implies that for our population the performance of the generated dynamic health policies is not extremely sensitive to the assumption of observability of new cases.

Now let us assume that the error term 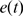 has the form 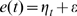, where 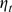 is a constant known to the policy maker and 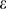 is a normally distributed noise with mean zero and standard deviation 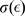. The effect of the constant term 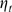 can be corrected like before by using equations 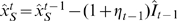 and 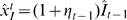 to update the current information about the epidemic state in Table 1. The effect of the random noise cannot be corrected; yet, the impact of this noise on the performance of the dynamic health policies can be investigated through simulation. Figure 6 shows the effect of uncertainty around the number of observed cases on the capability of dynamic health policies in controlling the epidemic in our population, for different WTP for health. We find that as the standard deviation of noise, 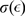, increases, the total number of individuals affected by the epidemic also increases; however, this increase is linear with a low rate which implies that for our population the performance of the generated dynamic health policies is not highly sensitive to the assumption of observability of new cases.

If this simulation analysis shows significant sensitivity of the performance of dynamic health policies to the assumption of observability of new cases, or the policy maker believes that the probability distribution of noise 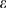 varies over time (for instance, if vigilance of testing diagnosis increases or decreases over time), then more advanced optimization tools can be employed. This issue is briefly discussed in the Discussion section.

## Discussion

The emergence of novel human pathogens (e.g. H1N1 and H5N1 influenza, SARS) and their devastating health and financial consequences on affected populations have highlighted the need for developing methods which allow real-time selection of health interventions to control the epidemic while effective vaccines are not available or not present in sufficient quantities to prevent disease spread. We refer to policies informed by such methods as *dynamic health policies* which are intended to allow for *real-time* recommendations to be made in response to changing disease and population characteristics as well as the availability of resources.

In contrast to most existing approaches for identifying optimal strategies for infectious disease control which use simulation or mathematical models of disease spread to compare the performance of a *limited* number of *pre-determined* health policies, we proposed the use of “dynamic programming” [23] to characterize and identify optimal dynamic health policies. We demonstrated how a Markov decision process [16] can be employed to find optimal dynamic health policies for a simple model of influenza epidemic, in which two types of interventions may be available during the epidemic to control the influenza spread: (1) vaccination, and (2) a transmission-reducing intervention, such as social distancing. The generated dynamic health policies help the policy maker to determine (1) how to allocate vaccines when they become available, and (2) whether the transmission-reducing intervention, such as school closure, should be employed or lifted given the number of susceptibles and infectives at any point of time.

While we used a discrete-time Markov decision process, a number of other methodologies have also been proposed and developed to determine or approximate optimal dynamic health policies for controlling emerging epidemic. For instance, Lefevre [24] used a continuous-time Markov decision model, Merl et al. [2] developed a statistical framework and Ludkovski and Niemi [21] developed a simulation-based model for dynamic determination of optimal policies for emerging epidemics. Undoubtedly, comparing the effectiveness of these methodologies when employed in real practice merits a separate research study.

The influenza model proposed in this paper is very simple and not intended to realistically model disease spread or be used directly to guide the selection of interventions. We present it only for illustration of our proposed approach for dynamic decision making. The model makes several simplifying assumptions that were required for using an MDP to generate the optimal health policies: first, we require that the number of new infections during each period is observable by the policy maker, and second, we assume that an infectious individual interacts with the rest of the population only during the next period and then is effectively removed (treated or isolated) from the population. Relaxing these two assumptions will mean the state of the epidemic is unobservable; yet, a probability *belief* about the epidemic state can often be generated as new data become available. If we relax these assumptions, we can use generalized discrete-time Markov models for infectious diseases proposed in [19] and use partially observable Markov decision process (POMDP) [25] to characterize optimal health policies. However, as the number of states required to model disease spread increases, MDP and POMDP rapidly lose their efficiency. In these cases, approximate dynamic programming [22] using a simulation model may be employed to identify optimal dynamic health policies. Accordingly, the framework we propose here can potentially be extended to inform decision-making for control of a pathogen with a more complex natural history such as tuberculosis or to design interventions that consider distinct responses targeted toward different risk groups. Such extensions are attractive topics for future research.

As a final note, for the successful implement of the dynamic health policies in practice, the mathematical or simulation model of the disease spread along with the optimization technique used for finding the dynamic health policies must be coupled with an surveillance system that can supply data to estimate the parameters of the underlying model and to provide knowledge on the state of the epidemic. Although dynamic optimization techniques are capable of handling noisy observations, inaccuracies in the surveillance and reporting system may result in suboptimal policies, further underscoring the tremendous importance of public health surveillance in defining responses to epidemics.

## Supporting Information

Text S1 **Calculating the Transition Probabilities for the Markov Model of Influenza Spread.** Approximating the State Space for Influenza Spread. Aditional Information for the Illustrative Example.
(PDF)
