# Supplementary material for: Dynamic Health Policies for Controlling the Spread of Emerging Infections: Influenza as an Example
Source: PLoS One. 2011 Sep 6;6(9):e24043. doi: 10.1371/journal.pone.0024043 (PMC3167826; doi:10.1371/journal.pone.0024043)
Supplement: Text S1 — Calculating the Transition Probabilities for the Markov Model of Influenza Spread. Approximating the State Space for Influenza Spread. Aditional Information for the Illustrative Example. (PDF) [file pone.0024043.s001.pdf]

Supplementary Information

Dynamic Health Policies for Controlling the Spread of Emerging Infections: Influenza as an Example

Reza Yaesoubi[1,2,*], Ted Cohen[1,2,3]

[1] Center for Communicable Disease Dynamics, Harvard School of Public Health, Boston, MA, U.S.A

[2] Division of Global Health Equity, Brigham and Women's Hospital, Boston, MA, U.S.A

[3] Department of Epidemiology, Harvard School of Public Health, Boston, MA, U.S.A

[*] E-mail: Corresponding reza.yaesoubi@gmail.com

Calculating the Transition Probabilities for the Markov Model of Influenza Spread

Using the framework provided in [1], the transition probabilities of the Markov chain $\{(X_S(t), X_I(t)) : t = 0, 1, \ldots\}$ can be calculated in 4 steps, which are outlined below. The reader is referred to [1] for detailed discussion of each step.

Step 1 - Define the classes and form the dynamics state equation:
For a population with fixed size N, the dynamics state equation will be $X_S(t) + X_I(t) + X_R(t) = N$; hence two classes are sufficient to construct the Markov model. We select $(X_S(t), X_I(t))$ as the state of the Markov model.

Step 2 - Find the joint probability distribution of the driving events:
There are two driving events in the model: (1) the number of new infections during period $[t, t + \Delta t]$, denoted by $I(t)$, and (2) the number recovered from infection during period $[t, t + \Delta t]$, denoted by $R(t)$. The probability distribution of the driving event $I(t)$ given the current state, $P_{I(t)}(i|X_S(t), X_I(t))$, is given by Eq. (5). As explained in the main manuscript, we assume that a susceptible who just became infected remains infectious during the next period, and then is removed from the population (or recovered). This assumption implies that any infective in the population at time t, will be removed at time $t + \Delta t$; hence, the probability distribution of the driving event $R(t)$ given the current state, $P_{R(t)}(r|X_S(t), X_I(t))$, will be:

$$P_{R(t)}(r|X_S(t), X_I(t)) = \begin{cases} 1, & \text{for } r = X_I(t), \\ 0, & \text{otherwise.} \end{cases} \tag{S1}$$

Step 3 - Form the dynamics driving and feasibility constraints:
Dynamics driving constraints for this model are as follows:

$$I(t) = X_S(t) - X_S(t + \Delta t), \tag{S2}$$

$$R(t) = X_I(t) - X_I(t + \Delta t) + X_S(t) - X_S(t + \Delta t). \tag{S3}$$

The dynamics feasibility constraints will be:

$$0 \le X_S(t) - X_S(t + \Delta t) \le X_S(t), \tag{S4}$$

$$0 \le X_I(t) - X_I(t + \Delta t) + X_S(t) - X_S(t + \Delta t) \le X_I(t). \tag{S5}$$

Since the joint probability distribution of the driving events $(I(t), R(t))$ is nonzero only if $R(t) = X_I(t)$ (by probability function (S1)), the dynamics driving and feasibility constraints (S2)-(S5) can be simplified as:

$$I(t) = X_S(t) - X_S(t + \Delta t) = X_I(t + \Delta t), \tag{S6}$$
$$0 \le X_S(t) - X_S(t + \Delta t) \le X_S(t). \tag{S7}$$

Step 4 - Calculate the transition probability of the Markov chain $\{(X_S(t), X_I(t)) : t = 0, 1, \ldots\}$:
By Eqs. (S6)-(S7), the support of the probability function

$$\Pr\{(X_S(t + \Delta t), X_I(t + \Delta t)) = (x_S, x_I)|X_S(t), X_I(t)\}$$

is calculated as:

$$\Omega_{(X_S(t),X_I(t))} = \{(x_S, x_I) \in \mathbb{N}^2|0 \le x_S \le X_S(t), 0 \le x_I \le X_S(t), x_S + x_I = X_S(t)\} \tag{S8}$$

Using the probability support $\Omega_{(X_S(t),X_I(t))}$ of Eq. (S8) and the fact that $I(t) = X_I(t+\Delta t)$ from Eq. (S6), the transition probability (6) is then obtained.

Approximating the State Space for Influenza Spread

For a population of size N, the transition probability matrix of the Markov chain $\{(X_S(t), X_I(t)) : t = 0, 1, \ldots\}$ is of size $0.5(N + 1)^2$, which for large populations causes computational problems for finding optimal health policies [2]. As a solution, we propose an approach for approximating the potentially enormous Markov chain $\{(X_S(t), X_I(t)) : t = 0, 1, \ldots\}$ with the Markov chain $\{(\Theta_S(t), \Theta_I(t)) : t = 0, 1, \ldots\}$, where $\Theta_C(t)$, $C \in \{S, I\}$ is the *proportion* of population in class $C \in \{S, I\}$ at time t, and can only take a limited number of values from the set $\{\theta^C_1, \theta^C_2, \ldots, \theta^C_{d_C}\}$, $C \in \{S, I\}$.

To determine the sets $\{\theta^C_1, \theta^C_2, \ldots, \theta^C_{d_C}\}$, $C \in \{S, I\}$, we note that by transition probability (6), the state of the disease at time t, i.e. $(X_S(t), X_I(t))$, restricts the feasible states which the disease may reach by time $t+1$; that is, the state $(X_S(t+\Delta t), X_I(t+\Delta t))$ should satisfy $X_S(t+\Delta t)+X_I(t+\Delta t) = X_S(t)$. Equivalently, for the approximate Markov chain $\{(\Theta_S(t), \Theta_I(t)) : t = 0, 1, \ldots\}$, we have:

$$\Theta_S(t + \Delta t) + \Theta_I(t + \Delta t) = \Theta_S(t). \tag{S9}$$

Therefore, the sets $\{\theta^C_1, \theta^C_2, \ldots, \theta^C_{d_C}\}$, $C \in \{S, I\}$ cannot be independently determined. For the proportion of susceptibles, let the *control points* $\{0 = b^S_0, b^S_1, b^S_2, \ldots, b^S_{d_S} = 1\}$, $0 \le b^S_1 < b^S_2 < \ldots < b^S_{d_S} = 1$, divide the interval $[0, 1]$ into d_S equal regions; that is $b^S_i = i/d_S$ for $i = 0, 1, \ldots, d_S$. The possible values of $\Theta_S(t)$, i.e. $\{\theta^S_1, \theta^S_2, \ldots, \theta^S_{d_S}\}$, are determined by $\theta^S_i = (b^S_{i-1} + b^S_i)/2 = (i - 1/2)/d_S$, for $i = 1, \ldots, d_S$. Thus, by Eq. (S9), $\Theta_I(t + \Delta t)$ can only take values from the set $\{\theta^S_i - \theta^S_j|i, j \in \{1, \ldots, d_S\}, j \le i\}$, which is equal to $\{i/d_S|i = 0, 1, \ldots, d_S - 1\}$. The control points for the proportion of infectives, i.e. $\{b^I_0, b^I_1, b^I_2, \ldots, b^I_{d_I}\}$, can be then determined by: $b^I_i = (i - 1/2)/d_S$ for $i = 1, 2, \ldots, d_S$ and $b^I_0 = 0$.

Given the state of the disease spread at time t, $(X_S(t), X_I(t))$, the support of the probability distribution (6) is given by the set $\Omega_{s_t} = \{(x_S, x_I)|x_I = X_S(t) - x_S, x_I + x_S \le N, x_I \ge 0, x_S \ge 0\}$; Figure S1 shows the support Ω_{s_t} for current state $(X_S(t), X_I(t))$, in which the set Ω_{s_t} includes the points lying on the bold line. Now, we must assign each point in the support Ω_{s_t} to the regions created by the control points $\{b^S_0, b^S_1, b^S_2, \ldots, b^S_{d_S}\}$ and $\{b^I_0, b^I_1, b^I_2, \ldots, b^I_{d_I}\}$ in order to create a valid probability transition matrix for the approximate Markov chain $\{(\Theta_S(t), \Theta_I(t)) : t = 0, 1, \ldots\}$. Table S1 shows an algorithm for calculating the transition probabilities for Markov chain $\{(\Theta_S(t), \Theta_I(t)) : t = 0, 1, \ldots\}$.

All the arguments presented in the main text for real-time decision making remain valid when the approximate Markov chains $\{(\Theta_S(t), \Theta_I(t)) : t = 0, 1, \ldots\}$ are used to model disease spread.

Additional Information for the Illustrative Example

To use the proposed class of models, we also must specify the control points $\{b_0^C, b_1^C, b_2^C, \ldots, b_{d_C}^C\}$, $C \in \{S, I\}$, dividing the interval $[0, 1]$ for the proportion of susceptibles and infectives. Control points for the proportion of susceptibles are set to $b_i^S = i/50$ for $i = 0, 1, \ldots, 50$, and as discussed above, control points for the proportion of infectives can then be determined accordingly: $b_i^I = (i - 1/2)/50$ for $i = 1, 2, \ldots, 50$ and $b_0^I = 0$.

Figures S3-S5 display three optimal health policies for three different scenarios for willingness-to-pay for health equal to \$0/QALY, \$25,000/QALY, and \$50,000/QALY.

References

1. Yaesoubi R, Cohen T (In Press) Generalized Markov models of infectious disease spread: A novel framework for developing dynamic health policies. European Journal of Operational Research .

2. Keeling MJ, Ross JV (2009) Efficient methods for studying stochastic disease and population dynamics. Theoretical Population Biology 75: 133–141.

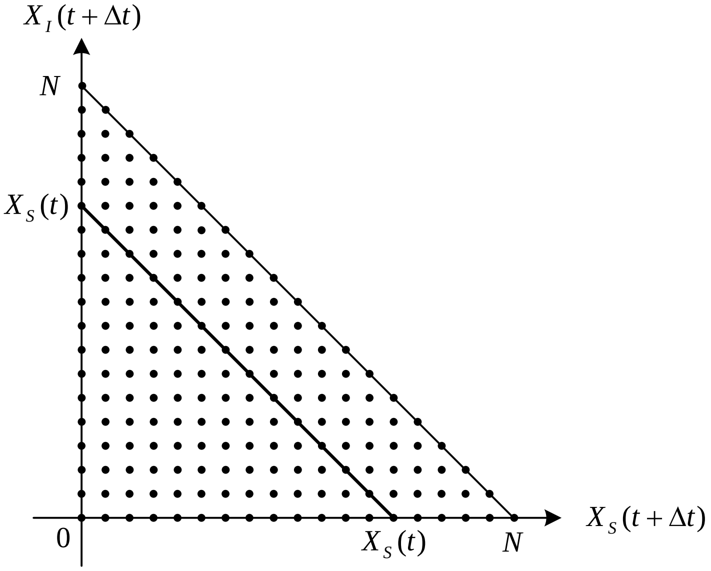

Figure S1. Support of the probability distribution (6)

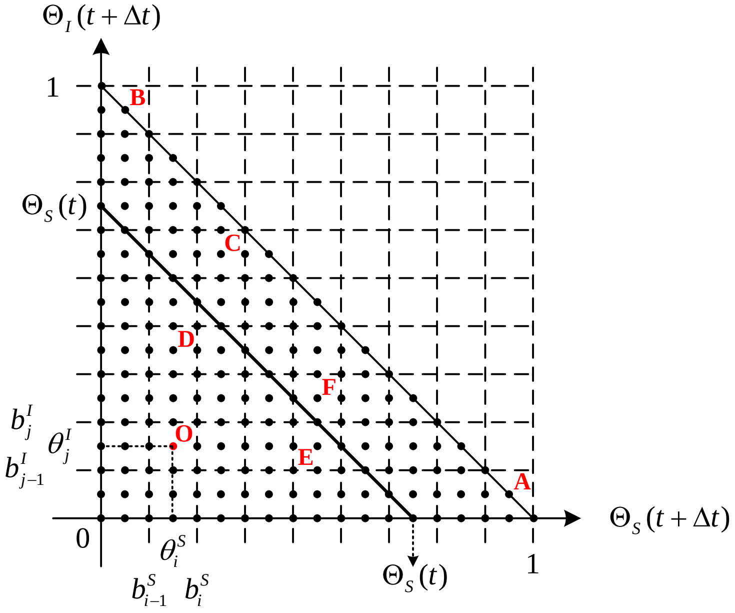

Figure S2. Calculating the support of probability $\Pr\{(\Theta_S(t+\Delta t), \Theta_I(t+\Delta t)) = (\theta_i^S, \theta_j^I) | \Theta_S(t), \Theta_I(t)\}$

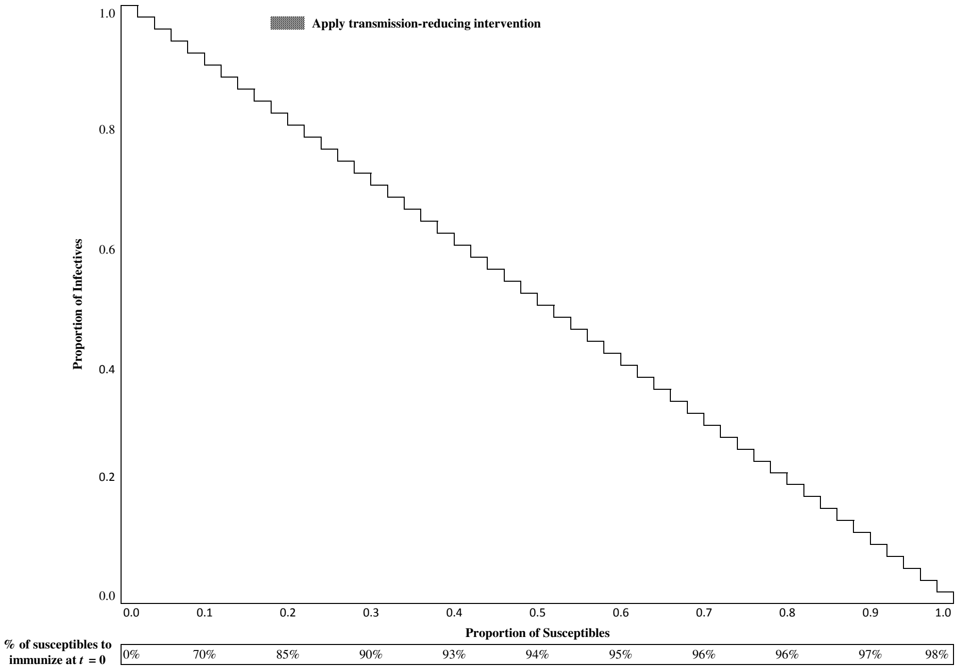

Figure S3. Optimal health policy for $\lambda = \$0/\text{QALY}$

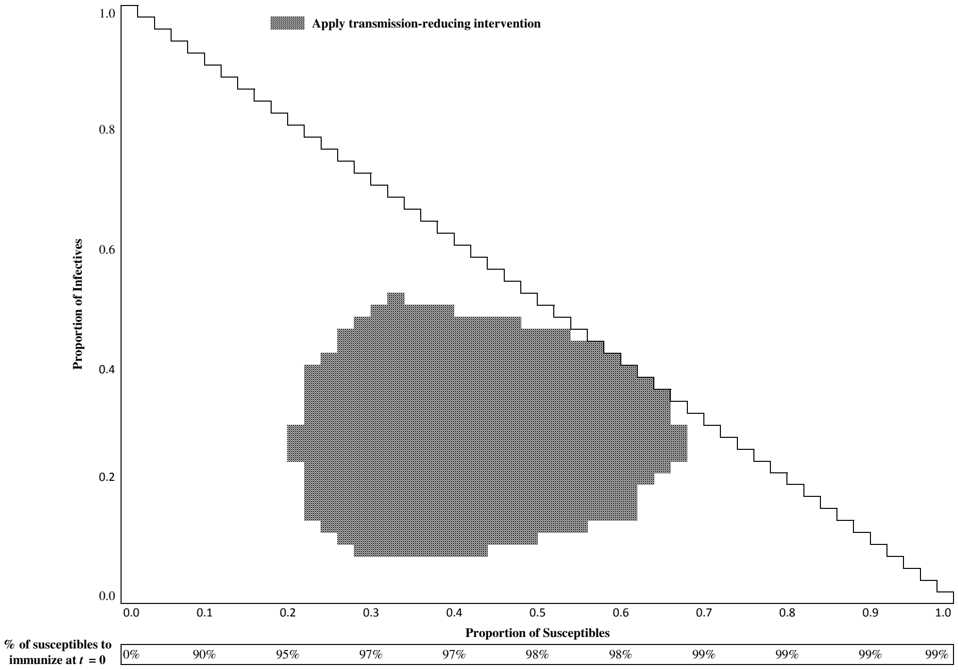

Figure S4. Optimal health policy for $\lambda = \$25{,}000$/QALY

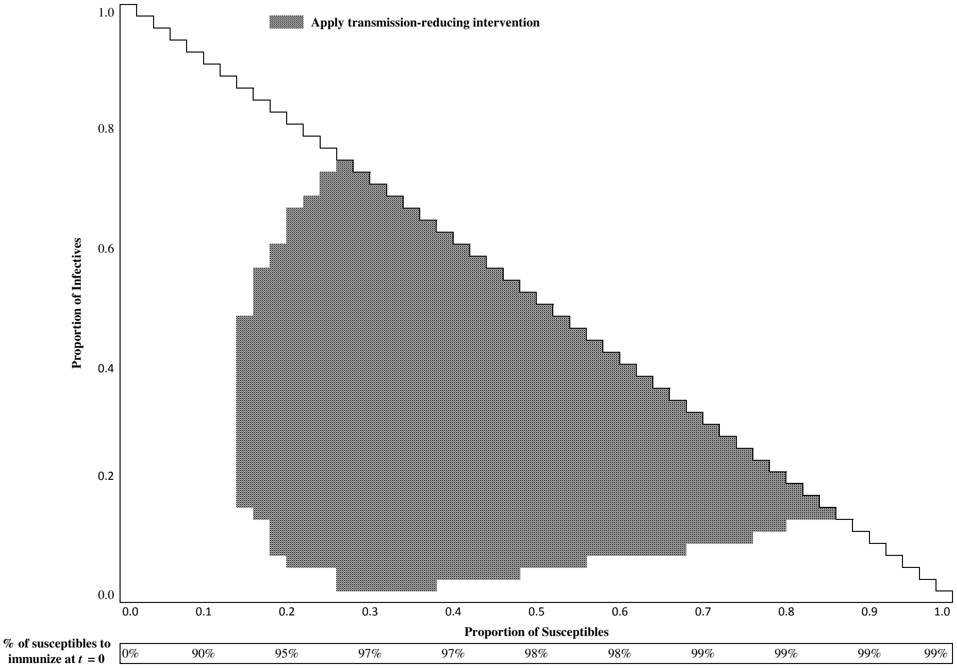

Figure S5. Optimal health policy for $\lambda = \$50{,}000/\text{QALY}$

Table S1. Calculating the transition probabilities for Markov chain $\{(\Theta_S(t), \Theta_I(t)) : t = 0, 1, \ldots\}$

Define operator $\lfloor \cdot \rfloor_l$ as:

$$\lfloor x \rfloor_l = \begin{cases} \lfloor x \rfloor - 1, \text{ if } x = \lfloor x \rfloor \\ \lfloor x \rfloor, \text{otherwise.} \end{cases}$$

// To calculate $\Pr\{(\Theta_S(t+\Delta t), \Theta_I(t+\Delta t)) = (\theta_i^S, \theta_j^I) | \Theta_S(t), \Theta_I(t)\}$: (See the red dot in region O in Figure S2.)

If $b_{i-1}^S + b_{j-1}^I \geq \Theta_S(t)$ **Or** $b_i^S + b_j^I \leq \Theta_S(t)$ **Then** // Region C and Region D respectively
    $\bar{x}_S \leftarrow 0$
    $\underline{x}_S \leftarrow 0$
Else If $X_S - Nb_{j-1}^I \leq \lfloor Nb_j^S \rfloor_l$ **Then** // Region F
    If $b_{j-1}^I = 0$ **Then**
        $\bar{x}_S \leftarrow X_S$
    Else
        $\bar{x}_S \leftarrow X_S - (\lfloor Nb_{j-1}^I \rfloor_l + 1) - 1$
    End If
    If $b_{j-1}^S = 0$ **Then**
        $\underline{x}_S \leftarrow 0$
    Else
        $\underline{x}_S \leftarrow \lfloor Nb_{j-1}^S \rfloor_l + 1$
    End If
Else // Region E
    $\bar{x}_S \leftarrow \lfloor Nb_j^S \rfloor_l$
    $\underline{x}_S \leftarrow X_S - \lfloor Nb_j^I \rfloor_l$
End If

$$\Pr\{(\Theta_S(t+\Delta t), \Theta_I(t+\Delta t)) = (\theta_i^S, \theta_j^I) | \Theta_S(t), \Theta_I(t)\} \leftarrow \sum_{x_S = \underline{x}_S}^{\bar{x}_S} P_{I(t)}(X_s(t) - x_S | X_s(t), X_I(t))$$
